# Assessment of difficult laryngoscopy by electronically measured maxillo-pharyngeal angle on lateral cervical radiograph: A prospective study

**DOI:** 10.4103/1658-354X.71572

**Published:** 2010

**Authors:** Kumkum Gupta, Prashant K. Gupta

**Affiliations:** *Department of Anesthesiology & Critical Care, N.S.C.B.Subharti Medical College, Subhartipuram, NH-58, Meerut, Uttar Pradesh, India*; 1 *Department of Radio-diagnosis, Imaging & Interventional Radiology, N.S.C.B.Subharti Medical College, Subhartipuram, NH-58, Meerut, Uttar Pradesh, India*

**Keywords:** *Maxillo-pharyngeal angle*, *laryngoscopy*, *cervical radiograph*, *electronic measurement of angle*, *Cormack and Lehane grade*

## Abstract

**Background:**

Difficult airway continued to be a major cause of anesthesia-related morbidity and mortality. Successful airway management depends on direct laryngoscopy and tracheal intubation. Difficult laryngoscopy is a resultant of incomplete structural arrangements during the process of head positioning. Through clinical history, examination of the patients along with craniofacial indices alerts the anesthetist for difficult laryngoscopy. But it does not predict all causes of difficult laryngoscopy during pre-anesthetic evaluation. The maxillo-pharyngeal angle, an upper airway anatomical balance, was proposed for better understanding the pathophysiology of difficult laryngoscopy. In our study we have assess difficult laryngoscopy by electronically measuring maxillo-pharyngeal angles on a lateral cervical radiograph. This angle is normally greater than 100°. Less than 90° angle suggests either impossible or difficult direct laryngoscopy when all known craniofacial indices were within the normal range. Cervical radiographic assessment is a simple, economical, and non-invasive predictive method for difficult laryngoscopy. It should be used routinely along with other indices as pre-anesthetic airway assessment criteria to predict the difficult laryngoscopy.

**Context:**

Difficulties with airway management continue to be a major cause of anesthesia-related morbidity, mortality, and litigation. Pre-operative assessment of difficult laryngoscopy by the simple and non-invasive radiological method can help to prevent them.

**Aims:**

To assess the difficult laryngoscopy pre operatively by a simple and non invasive radiological method by electronically measuring maxillo-pharyngeal angle on a lateral cervical radiograph and it’s correlation with Cormack and Lehane grading.

**Settings and Design:**

This is a controlled, nonrandomized, prospective, cohort observation study.

**Patients and Methods:**

The 157 adult consented patients of ASA grade I to III of either sex, scheduled for elective surgery under general anesthesia with endo-tracheal intubation, were studied. The patients with identified difficult airway indices were excluded from the study. The maxillo-pharyngeal angle was electronically measured on a lateral cervical radiograph and was correlated with ease or difficulty of laryngoscopy under general anesthesia. Their degree of laryngeal exposure according to Cormack and Lehane classification grade was also noted.

**Statistical Analysis used:**

We performed univariate analyses to evaluate the association between the covariates and direct laryngoscopy.

**Results:**

In 148 patients (94.28%), the maxillo-pharyngeal angle was more than 100°, in 7 patients (4.45%) it was less than 90°, and in 2 patients (1.27%) the M-P angle was less than 85° with normal craniofacial indices. When the MP angle was less than 90°, the direct laryngoscopy was difficult which could be compared with to Cormack and Lehane classification grade III and IV.

**Conclusions:**

Lateral cervical radiographic assessment should be used as pre-anesthetic airway assessment criteria to predict the difficult laryngoscopy as it is a simple, safe and non-invasive method.

## INTRODUCTION

Difficult airway continued to be a major cause of anesthesia-related morbidity and mortality. The studies on airway management were done to identify criteria to predict difficult laryngoscopy, as the tracheal intubation has become a routine part of general anesthesia. Successful intubation depends on direct laryngoscopy, done to visualize the vocal cords through the originally curved oral airway space. The direct line of vision from the mouth to the glottic opening is achieved by aligning the oral, pharyngeal, and laryngeal axis with some degree of flexion at the lower cervical spine and extension at the upper cervical spine, especially at the atlanto-occipital joint. Failure of these structural arrangements in response to direct laryngoscopy will result in difficult laryngoscopy.

Several preoperative airway assessment tests had been proposed singly or in various combinations to predict the patients with difficult airway but few patients remain undetected despite the most careful preoperative airway evaluation. Predicting difficulty is being done by history of associated diseases, symptoms of airway compromise or physical examination revealed restricted mouth opening, jaw, or neck movements. The Mallampati classification reliably predicted difficult intubation which could be compared with the degree of laryngeal exposure according to Cormack and Lehane classification.

Unanticipated difficult laryngoscopy in patients for elective surgical procedures can occur due to the combination of several minor physical anomalies when no single factor is severely abnormal. A number of studies have attempted to combine physical factors to predict difficult intubation with mixed results. The maxillo-pharyngeal angle, an upper airway anatomical balance, was proposed for better understanding the pathophysiology of difficult laryngoscopy.

The objective of our study was to explore a simple, reproducible, and non-invasive radiological method to predict the difficult laryngoscopy preoperatively.

## PATIENTS AND METHODS

This nonrandomized prospective study was taken up as exploratory feasibility study to build an appropriately powered trial for the future. There were no previous studies to determine sample size. After approval of our Institutional Ethical Committee, the study was carried out with 157 adult consented patients in the age group of 15-65 years of both sexes scheduled for elective surgeries under general anesthesia with tracheal intubation. Patients with established craniofacial anomaly and those primarily scheduled for fiberoptic intubation were excluded from this study.

### Historical aspect

A patient was posted for mastoidectomy under general anesthesia. During pre-anesthetic evaluation, his airway assessment was judged within normal limits. After premedication and induction, tracheal intubation could not be performed successfully even at second attempt by routine method. Retrospectively this patient was investigated for cause of difficult laryngoscopy and intubation. The anesthetic literature was reviewed. One reference[1] quoted that if maxillo-pharyngeal angle, measured on lateral cervical radiograph, is less than 90° with other normal craniofacial parameters, it would be a difficult laryngoscopy. It was found to be true for this patient. After this incidence, the lateral cervical radiograph was included in pre anesthesia check up list to reduce the risk of difficult laryngoscopy and airway.

### Exclusion criterion

All patients with any airway-related problems such as massive obesity, cervical collars, traction devices, and external trauma were not included in the study. The patients with restricted mouth opening function of the tempero-mandibular joint, long and narrow mouth with a high-arched palate, the short, thick and muscular neck, neck masses and fixation of the trachea may pose difficult laryngoscopy, were also excluded from the study. The presence of ear and hand anomalies often suggested the presence of difficult airway.

### Maxillo-pharyngeal angle

According to the definition described by Adnet *et al*.,[2] the line parallel to the hard palate is known as the maxillary axis (MA) while the line passing through the anterior portion of the first (atlas) and second cervical vertebra is known as pharyngeal axis (PA). The angle between the MA and the PA is defined as maxillo-pharyngeal angle.

### Radiological measurements

**Figure 1:**
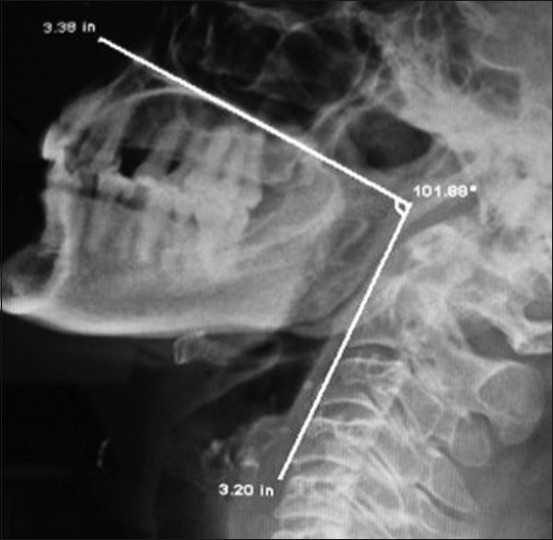
Radiograph of Neck-Lateral view showing the normal maxillo-pharyngeal angle > than 100 degree (Source: Department of Radio-diagnosis, Imaging and Interventional Radiology N.S.C.B.Subharti Medical College, Meerut)

A lateral cervical radiograph was taken in erect posture of patients with the neutral position of head. Before exposure, the patients were instructed to close the jaw in the natural occlusive position and to breathe quietly. The radiograph was taken at the end of expiration. The radiological exposure parameters were arranged to clearly visualize bony landmarks. The cervical radiographs analysis was done by experienced radiologist electronically, unaware of the study. Anatomical landmarks were identified and connected for the purpose of angle measurements. The MA and PA were defined by the line parallel to the hard palate and the line passing through the anterior portion of the first cervical vertebra (atlas) and of second cervical vertebra, respectively. The angle between the MA and PA was defined as the maxillo-pharyngeal angle. Normally the maxillo-pharyngeal angle is greater than 100° [Figure 1].

Mallapati classification and airway assessment were done during pre-anesthesia check up. Their Cormack and Lehane grading for laryngeal exposure was also done during direct laryngoscopy under general anesthesia. All these parameters were studied together to formulate predictors of difficult laryngoscopy.

## RESULTS

Pre-operative assessment for air way evaluation was performed by examining the mouth opening, oral cavity, and neck movements. The craniofacial dimensions were found to be within the limits of normal in all patients.

Modified Mallampatti classification and Cormack and Lehane grading were correlated along with atlantooccipital (A-O) joint extension, thyromental (T-M) distance and maxillo-pharyngeal (M-P) angle. The radiological measurement of maxillo-pharyngeal angle was done on lateral cervical radiograph for prediction of difficult laryngoscopy pre-operatively [Table 1].

**Table 1:** Correlation between different parameters

Mallampati classfication	Cormack and Lehane	M-P angle	A-O extension	T-M distance
I	I	>110°	>35°	>6.5 cm
II	II	<110-90°	30-34°	6-6.5 cm
II	III	<90°	25-29°	6-6.5 cm
II	IV	<85°	<25°	<6 cm

M-P: Maxillo-pharyngeal

**Figure 2:**
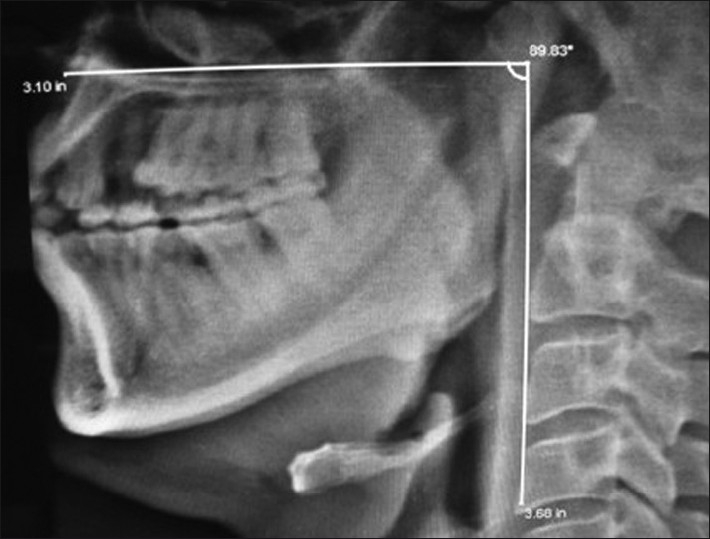
Radiograph of neck - Lateral view showing the abnormal maxillo-pharyngeal angle < than 90 degree (Source: Department of Radio-diagnosis, Imaging and Interventional Radiology N.S.C.B.Subharti Medical College, Meerut)

We found that when M-P angle was between 110° and 100°, direct laryngoscopy could be performed easily in148 patients (94.28%) and when M-P angle was less than 90°, it was impossible to visualize the larynx at direct laryngoscopy [Figure 2]. In our study, the overall proportion of patients with difficult laryngoscopy was 5.72% [Table 2].

**Table 2:** Percentage distribution of patients

Parameters	M-P angle	No. of patients	Percentage
	>110°	102	64.96
	110-90°	46	29.29
	<90°	07	4.45
	<85°	02	1.27

## DISCUSSION

Difficult laryngoscopy, resultant of incomplete structural arrangements during the process of head positioning, is a multifactorial problem and no single test can predict difficulty accurately. Unanticipated difficult laryngoscopy in patients for elective surgical procedures can occur due to the combination of several minor physical anomalies, when no single factor is severely abnormal. A number of studies have attempted to combine physical factors to predict difficult laryngoscopy but none could succeed over the clinical evaluation. The existing predictors of difficult laryngoscopy are not sensitive or specific enough for routine use.

Direct laryngoscopy is performed to visualize the vocal cords through the originally curved oral airway space by aligning the oral, pharyngeal, and laryngeal axis in morning sniffing position of head. The simple neck extension and the sniffing position produced a caudal shift of the mandible and a downward shift of the larynx, resulting in an increase of the submandibular space. An increase in the sub-mandibular space and a vertical arrangement of the mandible, tongue base, and larynx to the facial line are important mechanisms for improving the lar yngeal view during direct laryngoscopy.[3] The BURP maneuver, which includes backward, upward, and right lateral displacement of the thyroid cartilage, has been shown to be effective in improving the grade of glottic exposure.[4]

The prediction of difficult laryngoscopy is not always an easy task. In certain patients, the causes are obvious such as the facial deformity, abnormal limitation of the temporomandibular joint movement,[5] hypopharyngeal disease, limited neck extension (<35°), a distance between the tip of the patient’s mandible and hyoid bone of less than 7 cm, a sternomental distance of less than 12.5 cm with the fully extended head and mouth closed, and a poorly visualized uvula during voluntary tongue protrusion.[6] Obesity may create anatomical difficulties for the laryngoscopy caused by the decreased mobility and enlargement of structures in the throat and around the neck.[7] Cervical trauma, cervical arthritis, or previous cervical fusion may restrict the desired sniffing position of head. These were established causes of difficult layrngoscopy and these patients are at risk of difficult airway.

When the patients presented with obvious lesions, established tests or criteria should be used only as indicators. Interesting results were obtained when radiological criteria were used.[8] In some studies, the use of ultrasonography has been suggested to anticipate the degree of airway difficulty, but this technology cannot be advocated for routine clinical practice.[9]

Mallampati and colleagues emphasized the importance of the base of the tongue in determining the difficulty of laryngoscopy[10] which is a simple, reproducible, and reliable pre-anesthetic airway assessment method but has limited discriminative power for difficult tracheal intubation. Upper bite test was proposed as an alternative to the widely used test, the modified Mallampati classification[11], which reliably predicted difficult intubation and could be compared with Cormack and Lehane classification for the degree of laryngeal exposure during laryngoscopy.[12]

Several workers have used multivariate risk indices with or without scoring to enhance the sensitivity of predicting difficult intubation.[13] The incidence of difficult laryngoscopy (1.5%-13%), difficult intubation (1.2%-3.8) and difficult mask ventilation (.01%-.05%) are subject to anesthesiologist variability but they do occur. Shiga *et al*. indicates that direct laryngoscopy can be difficult in 5.8% of the general anesthesia population.[14] Lewis *et al*. conducted a study to determine which method of testing predicts difficult laryngoscopy[15] Even calculating airway indices that incorporate many assessment criteria, those proposed by Wilson *et al*.[16] and Arne *et al*.,[17] did not consistently ensure accurate evaluation of difficult laryngoscopy. Wilson scoring test has a sensitivity of 75%, specificity of 88%, and positive predictive value of 9% and negative predictive value of 99%. Mallampati grading was combined with factors such as obesity, short neck, abnormal teeth, receding mandible, facial edema, and swollen tongue in the obstetric population and showed a significant correlation between classification of airway and laryngoscopic grade. White and Kander have shown that the posterior depth of the mandible i.e. the distance between the bony alveolus immediately behind the third molar tooth and the lower border of the mandible was an important measure to determine the ease or difficulty of laryngoscopy.[18] The effective mandibular length was compared with the posterior depth of the mandible and the ratio of >3.6 indicates difficult intubation.

The variable results in all these studies was due to difficult intubation being uncommon and so none of the predictors has been able to yield a high positive predictive value for difficult laryngoscopic intubation.

In our study, the maxillo-pharyngeal angle, an upper airway anatomical balance, was proposed for better understanding the pathophysiology of difficult laryngoscopy. The radiological assessment was done by electronically measuring maxillo-pharyngeal angle on lateral cervical radiograph with head in the neutral position. The craniofacial indices were within normal limits in all the patients included in our study. It was observed that when maxillo-pharyngeal angle was greater than 100°, direct laryngoscopy could be easily performed, but when angle was less than 90°, it was impossible to visualize the larynx at laryngoscopy. It could be comparable with Cormack and Lehane III and IV grades of laryngoscopic view. Although these observation and measurements do not always predict difficult laryngoscopy, they are useful in approximately 80% of patients. These data may be obtained at the bed side to augment the history and clinical examination.

Though the ventilation can often be achieved in one way or other using supraglottic devices, even when direct laryngoscopy and tracheal intubation turn out to be impossible, but it is important to use a systemic approach and well defined plan, when faced with a patient with a potentially difficult airway.

### Limitation of study

Our structural analysis was two dimensional and did not include the whole upper airway structures possibly involved for difficult laryngoscopy, indicating a methodological drawback for investigation. Nevertheless it was surprising to find upper airway anatomical imbalance despite the methodological limitation while it ignores numerous factors that may influence pharyngeal airway patency. In reality, it was difficult to obtain numerous control patients from the general adult population.

## CONCLUSION

No single airway test can provide a high index of sensitivity and specificity for prediction of difficult laryngoscopy. Therefore it has to be a combination of multiple tests. The combination of modified Mallampati classification and measurement of maxillo-pharyngeal angle on lateral cervical radiograph in parallel is more sensitive and specific with clinically relevant higher discriminative power. The ability to predict difficult laryngoscopy preoperatively allows anesthesiologists to take precautions to reduce the anesthesia-related risks. This study adds to the numerous studies dealing with the prediction of difficult laryngoscopy.
